# Fragmentation Through Polymerization (FTP): A new method to fragment DNA for next-generation sequencing

**DOI:** 10.1371/journal.pone.0210374

**Published:** 2019-04-01

**Authors:** Konstantin B. Ignatov, Konstantin A. Blagodatskikh, Dmitry S. Shcherbo, Tatiana V. Kramarova, Yulia A. Monakhova, Vladimir M. Kramarov

**Affiliations:** 1 All-Russia Institute of Agricultural Biotechnology, Russian Academy of Sciences, Moscow, Russia; 2 Vavilov Institute of General Genetics, Russian Academy of Sciences, Moscow, Russia; 3 Evrogen JSC, Moscow, Russia; 4 Pirogov Russian National Research Medical University, Moscow, Russia; 5 The Department of Molecular Biosciences, The Wenner-Gren Institute, Stockholm University, Stockholm, Sweden; 6 Syntol JSC, Moscow, Russia; University of Helsinki, FINLAND

## Abstract

Fragmentation of DNA is the very important first step in preparing nucleic acids for next-generation sequencing. Here we report a novel Fragmentation Through Polymerization (FTP) technique, which is a simple, robust, and low-cost enzymatic method of fragmentation. This method generates double-stranded DNA fragments that are suitable for direct use in NGS library construction and allows the elimination of the additional step of reparation of DNA ends.

## Introduction

Next Generation Sequencing (NGS) has become one of the most widely used techniques in genomic research and genetic diagnostics. Fragmentation of DNA is the first main step in preparing a sequencing library for NGS. The well-known NGS technologies—like Illumina or Ion Torrent—generate a plethora of reads with lengths under 600–1000 bases. For library preparation, purified DNA samples are sheared into shorter fragments, then platform-specific adapters are ligated to the molecules to provid primer-binding sites for further amplification and sequencing. The high level of NGS resolution is achieved by multiple representations through different reads for every DNA region despite their sequence and context. In other words, the sequences of the fragments must overlap. Thus, the quality of NGS is largely dependent on the randomness of DNA fragmentation and the overlap of the resulting library fragments. This makes the fragmentation step critical in the process of library construction.

There are three typical approaches to shorten long DNA for library preparation: physical (by using acoustic sonication or by hydrodynamic shearing), enzymatic (based on the usage of endonucleases or transposase) and chemical shearing (by hydrolyzing DNA through heating it with divalent metal cations) [1, 2].

Acoustic shearing with Covaris ultrasonicators (Covaris, Woburn, MA, USA) is currently the gold standard for fragmentation at random nucleotide locations for an NGS library construction; this process is very important for a high-quality NGS library sample preparation. Unfortunately, it can be financially inaccessible for many laboratories [3]. An additional disadvantage of acoustic shearing is that it can be a source of oxidative damage to DNA that may result in sequencing artifacts [4].

Enzymatic methods and acoustic shearing have similar levels of efficiency, but enzymatic methods do not need expensive equipment [2]. Commercially available Fragmentase (New England Biolabs, Ipswich MA, USA) and Nextera tagmentation (Illumina, San Diego, CA, USA) are the most popular enzymatic techniques. Nextera uses a transposase to simultaneously fragment and insert adapters into dsDNA [5]. Fragmentase contains two enzymes: one randomly nicks dsDNA and the other cuts the strand opposite to the nicks [2]. Enzymatic digestion is simple and very efficient, but it may introduce an enzymatic bias, such as insertions and deletions (indels) [2, 6]. These biases are associated with DNA sequence content and may produce a non-random fragmentation [6].

**Fig 1:**
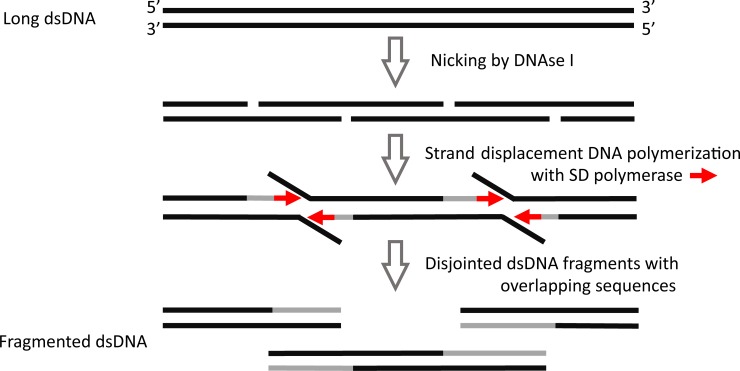
A general overview of the dsDNA Fragmentation Through Polymerization (FTP) method. The FTP method is based on two enzymatic reactions: a DNA nicking reaction with DNase I and a strand-displacement DNA polymerization with SD DNA polymerase. As a result, multiple double-stranded DNA fragments with overlapping sequences are generated. *De novo* synthesized DNA is indicated in grey, and SD polymerase is indicated in red.

DNA fragments obtained by physical fragmentation or by the Fragmentase method require a repair of DNA ends for the ligation with adapters during subsequent NGS library construction [1, 2]. To improve the protocol for NGS library generation and reduce the end repair stage, we have developed a new enzymatic method for DNA fragmentation: Fragmentation Through Polymerization (FTP). Our FTP method is based on the use of two enzymes: a non-specific endonuclease, which randomly nicks dsDNA (DNase I), and a thermostable DNA polymerase with strong strand-displacement activity (SD DNA polymerase) [7]. At the first stage of FTP, DNase I introduces nicks into the dsDNA, and at the second stage, SD DNA polymerase elongates the 3’-ends of the nicks in a strand-displacement manner. As a result, FTP generates multiple double-stranded DNA fragments with extended overlapping sequences at the ends (Fig 1). Additionally, the SD polymerase causes 3’-A-overhangs, which make the fragments suitable for direct ligation with T-tailed DNA adapters without a requiring DNA end repair.

A random fragmentation process is an important feature for high-quality NGS library sample preparation. It is known that DNA cleaving is not an entirely random process because cleaving/nicking enzymes—including DNase I—are sequence-dependent [8, 9], and physical methods for fragmentation are partly sequence-specific as well [10, 11]. Like other enzymatic methods, FTP utilizes DNase I as a nicking enzyme. In contrast to other digesting techniques, the fragments obtained by FTP from a long DNA molecule have overlapping sequences at the ends (Fig 1) that may help to overcome the problem with sequence-dependent DNA-nicking by DNase I.

Here we describe the detailed FTP method of DNA fragmentation and compare it with the well-known and widely used Fragmentase technique (New England Biolabs). Systematic comparison of Fragmentase with other fragmentation methods has been described earlier [2].

## Materials and methods

### Enzymes and reagents

Lyophilized DNase I (deoxyribonuclease I from Bovine pancreas) was obtained from Sigma-Aldrich (St Louis, MO, USA) and dissolved in the storage buffer (50% glycerol, 100 mM NaCl, 0.2 mg/ml BSA, 1 mM EDTA, 0.2 mM DTT, 20 mM Tris-HCl, pH = 8.0) up to 1 mg/ml.

SD DNA polymerase (50 U/μl) and the reaction buffer were supplied by Bioron GmbH, (Ludwigshafen, Germany). *E*.*coli* BL21(DE3) gDNA was supplied by Evrogen JSC (Moscow, Russia). dNTPs were obtained from Bioline Limited (London, UK).

NEBNext dsDNA Fragmentase and the NEBNext Ultra II DNA Library Prep kit were supplied by New England Biolabs, Inc. (Ipswich, MA, USA).

### dsDNA Fragmentation Through Polymerization (FTP)

For fragmentation, 200 ng of gDNA of the *E*. *coli* strain BL21(DE3) were added to the following reaction mixture: 1X reaction buffer for SD polymerase (Bioron GmbH), 3.5 mM MgCl_2_, 0.25 mM dNTPs (each), DNase I 1 ng/μl, SD DNA polymerase 1.5 U/μl. The total volume of the reaction was 25 μl. The reaction mixture was completed at 4°C (wet ice). The fragmentation of gDNA was carried out by two-step incubation: 20 minutes at 30°C and then 20 minutes at 70°C. For incubation, we used a thermal cycler with a heated lid. The reaction was stopped by cooling down the mixture to 10°C. The mixture was diluted 1:1 with sterile water, and fragmented DNA was purified with SPRI beads.

### DNA fragmentation with NEBNext dsDNA Fragmentase

gDNA of the *E*. *coli* strain BL21(DE3) was digested using NEBNext dsDNA Fragmentase (New England Biolabs, Inc.) according to the manufacturer’s protocol. Briefly, 200 ng of gDNA were added to the following reaction mixture (total volume 25 μl):1X Fragmentase Reaction Buffer v2, 10 mM MgCl, and 1X dsDNA Fragmentase. The mixture was incubated at 37°C for 20 minutes. The digestion was stopped by adding EDTA up to 100 mM. The mixture was diluted 1:1 with sterile water and fragmented DNA was purified with SPRI beads.

### Preparation of NGS libraries

We prepared four NGS libraries from four different samples of Fragmentase-digested gDNA and four NGS libraries from four different samples of FTP-digested gDNA. NGS libraries were generated using NEBNext Ultra II DNA Library Prep kit (New England Biolabs, Inc.) according to the manufacturer’s instructions. The conventional procedure for Fragmentase-digested DNA included repair of DNA ends with the NEBNext Ultra II End Prep Enzyme Mix, addition of adapters to the DNA fragments using NEBNext Ultra II Ligation Master Mix, and amplification of the adaptor-ligated DNA fragments with the NEBNext Ultra II Q5 Master Mix. The input amount of each DNA sample was 200 ng. The library indexing and amplification were performed for 5 PCR cycles as described in the kit’s manual.

NGS libraries from FTP digested gDNA were constructed using the NEBNext Ultra II DNA Library Prep Kit procedure, excluding the DNA end repair stage. The input amount of each DNA sample was 200 ng. The library indexing and amplification were performed for 5 PCR cycles with the NEBNext Ultra II Q5 Master Mix.

After the amplification stage, all libraries were quantified with a Quant-iT PicoGreen dsDNA Assay Kit (Molecular Probes, Inc., Eugene, OR, USA) and with the Agilent 2200 TapeStation Instrument with a D1000 Tape System (Agilent Technologies, Waldbronn, Germany), pooled (500 ng of each), and purified with AMPure XP beads.

### NGS and bioinformatic analysis

The pooled libraries were sequenced with the Illumina MiSeq Instrument (Illumina, California, USA) with a 300 Cycles MiSeq Sequencing Kit v2—paired-end mode—resulting in 12×10^6^ reads. Each of the reads was approximately 150 nt long. The FASTQ files generated on the instrument were uploaded to the NCBI SRArchive under project ID: PRJNA509202.

The FASTQ files were quality controlled using FASTQC v0.11.4 (Babraham bioinformatics, Cambridge, UK). PHRED scores were calculated with FASTQC v0.11.4. Adapters were trimmed with FLEXBAR v.2.5 [12]. Filtered reads with a minimum length of 30 bp were subsequently aligned to the *E*.*coli* BL21(DE3) genome (NCBI Reference Sequence: NC_012971.2) using BOWTIE2 software v2.3.4 [13]. For coverage uniformity evaluations, the Lorenz curves were built with the htSeqTools R package version 1.30.0 (https://rdrr.io/bioc/htSeqTools/) and the GC bias plots were obtained with the CollectGcBiasMetrics (Picard tools) software (https://software.broadinstitute.org/gatk/documentation/tooldocs/4.0.1.0/picard_analysis_CollectGcBiasMetrics.php). Random samples of reads were generated using Seqtk software (https://github.com/lh3/seqtk). *De novo* assembly of contigs was carried out with the SPAdes tool v3.10.1 (http://cab.spbu.ru/software/spades/). Statistics were calculated using QUAST software v5 [14, 15] (http://quast.sourceforge.net/).

## Results and discussion

### Digestion of gDNA with the FTP method

We compared two enzymatic methods of dsDNA fragmentation for NGS library construction: digestion with Fragmentase from New England Biolabs and FTP. The FTP method consists of two enzymatic reactions: random DNA nicking and elongation in a strand-displacement manner of the 3’ ends of the nicked DNA. As a result, multiple double-stranded DNA fragments with overlapping sequences at the ends are generated. The general overview of the FTP method is outlined in Fig 1.

We carried out FTP in a one-tube format as described above. Mesophilic DNase I and thermophilic SD DNA polymerase were added to the reaction mixture that contained the gDNA of the *E*. *coli* strain BL21(DE3). The reaction was incubated at 30°C for 20 minutes, plus an additional 20 minutes at 70°C. DNase I has an optimum performance temperature between 30°C and 40°C. During the first stage of incubation at 30°C, DNase I introduced nicks into the dsDNA. In order to optimally obtain average-sized fragments, we tested different DNase I concentrations and incubation times (S1 Fig). During the second stage, the DNase I was heat-inactivated and the SD polymerase was activated by increasing the reaction temperature to 70°C. The SD polymerase is a Taq DNA polymerase mutant that has a strong 5’–3’ strand displacement and 5’–3’ polymerase activities [7]. It does not have 5’–3’ and 3’–5’ exonuclease activities. Unlike natural enzymes with strong strand displacement activity, such as Phi29 or Bst polymerase that are stable and active below 70°C, SD polymerase is stable up to 93°C and has its optimum level of enzymatic activity at 70–75°C. Additionally, the enzyme does 3’-A-overhangs, which make the product of its polymerization suitable for ligation with T-tailed DNA adaptors. These properties of SD DNA polymerase make it very suitable for the FTP technique.

**Fig 2:**
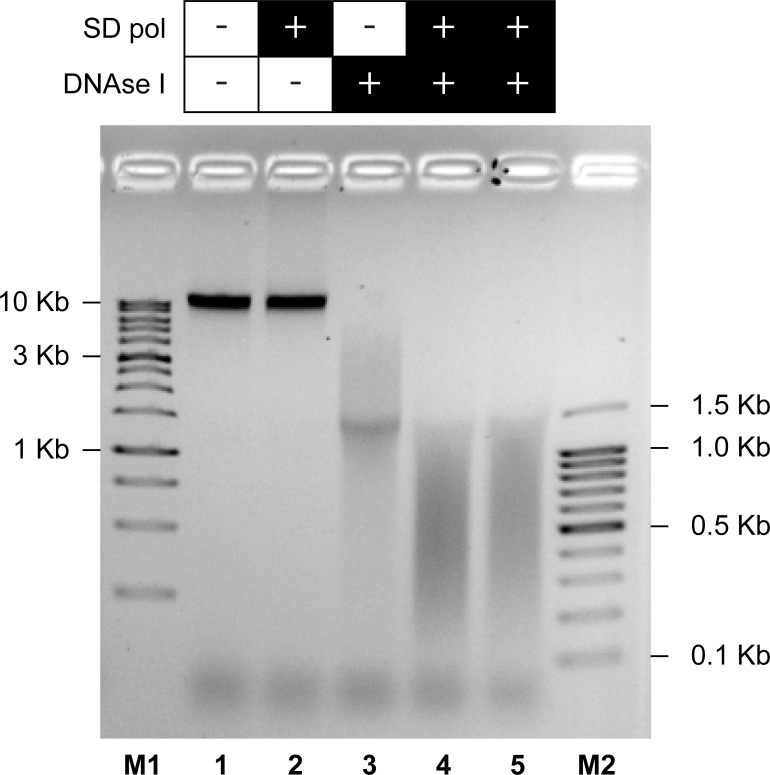
Agarose-gel electrophoresis of gDNA fragmented by the FTP method. gDNA of *E*. *coli* BL21 was incubated as described in Materials and Methods: without enzymes (lane 1), with SD polymerase (lane 2), with DNase I (lane 3), and with both DNase I and SD polymerase (lane 4 and 5). M1: 1 kb DNA Ladder; M2: 100 bp DNA Ladder.

In summary, DNase I generated 3’ ends by nicking dsDNA at 30°C, followed by SD polymerase using these ends for strand displacement DNA polymerization at 70°C, which resulted in disjointed dsDNA fragments (Fig 1). As the result, the A-tailed dsDNA fragments with overlapping sequences and with an average size of about 500 bp (in a range from 150 to 1500 bp) were obtained from the intact gDNA. Agarose-gel electrophoresis of gDNA fragmented by FTP is demonstrated in Fig 2. As seen in this figure, both DNase I and SD polymerase are required for the DNA fragmentation and complete separation of the fragments (Fig 2, lanes 4 and 5).

Fragmentase and other methods of fragmentation—with the exception of Illumina’s Nextera tagmentation—generate DNA fragments by introducing nicks and counter nicks in DNA strands that disassociate at 8–12 nucleotides downstream or upstream from the nick site. Thus, the generated fragments need repair of DNA ends for the subsequent NGS library construction [1, 2]. Unlike in other methods, in FTP the DNA fragments are separated by strand-displacement DNA polymerization and not by counter nicks. SD polymerase also carries out A-tailing of the ends. As a result of FTP, double-stranded DNA fragments have ends that are suitable for direct NGS library construction and the additional step of DNA end repair is no longer necessary.

### NGS library constructions from Fragmentase and FTP -digested gDNA

Two techniques—FTP and standard Fragmentase—were used to digest the gDNA of the *E*. *coli* strain BL21(DE3). The fragmented DNA samples were then used for the construction of NGS libraries with NEBNext Ultra II DNA Library Prep Kit from New England Biolabs. Four libraries were prepared from the DNA samples digested with Fragmentase by the standard protocol, which included the stage of DNA end repair.

Another four libraries were prepared using the same NEBNext kit, but the DNA samples for these libraries were generated with the FTP method without the stage of DNA end repair. It is worth noting that when the DNA fragments are obtained by physical fragmentation or from the Fragmentase method, the repair of the DNA ends is necessary for the library’s construction [1, 2]. The FTP method does not require this step; therefore, the procedure of NGS library preparation is simpler. As mentioned above, FTP generates A-tailed DNA fragments which are suitable for direct ligation with T-tailed adaptors. As a result, the preparatory time for NGS library creation has decreased by 70 minutes—from 180 minutes (the preparation with the end repair stage) to 110 minutes (without the stage of end repair).

The DNA amount in each library was quantified with the Quant-iT PicoGreen dsDNA Assay Kit and with the Agilent 2200 TapeStation. All NGS libraries generated with both the Fragmentase and the FTP method contained similar amounts of ds DNA (800 ± 50 ng) and had similar mean insert sizes of the libraries in a range from 400 to 500 bp. This result shows that the yield of the NGS libraries generated with the FTP method is comparable to the yield obtained with the Fragmentase technique.

### Assessment of NGS libraries generated from Fragmentase and FTP -digested gDNA

The NGS libraries of *E*. *coli* BL21(DE3) gDNA were sequenced at 48× depth with an Illumina MiSeq Instrument. The raw data (about 220 Mb for each DNA sample) generated in this study have been deposited in the National Center for Biotechnology Information (NCBI) Sequence Read Archive under BioProject accession number PRJNA509202 (https://www.ncbi.nlm.nih.gov/sra/PRJNA509202).

Different fragmentation and NGS library preparation protocols could potentially affect the quality of the reads. We therefore estimated the quality of reads as described in [2] for comparison of different fragmentation methods. PHRED quality scores for each base provide a sequencing error estimate and are a good tool to assess the quality of sequences and to compare the reliability of different sequencing runs on the same instrument [16]. We did not detect any significant differences in the quality scores obtained from the Fragmentase and FTP NGS libraries (S2 Fig).

**Fig 3:**
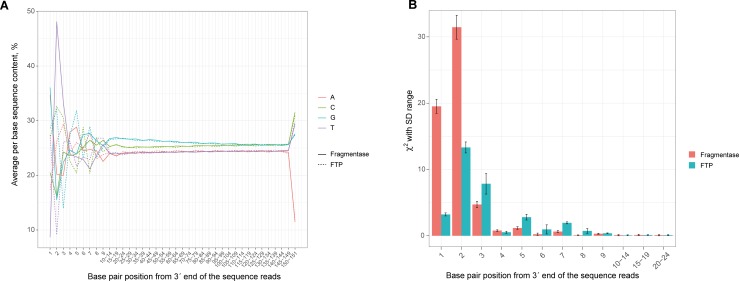
Comparison of the mean nucleotide compositions in the reads of FTP- and Fragmentase-generated NGS libraries. (A) Bias plots showing mean (by replication) percentage of observed bases at each position of reads for Fragmentase (solid lines) and FTP (dotted lines) methods of fragmentation. (B) χ^2^ values of observed bases at each position of reads (calculated by Pearson χ^2^ test for given probabilities) for Fragmentase (red) and FTP (blue) methods. Given probabilities are mean probabilities for each nucleotide from position 11 to position 149. Smaller χ^2^ values indicate that the observed probability of bases at the given position is closer to the mean probabilities at the non-bias region and less bias is observed.

The randomization of DNA digestion for both fragmentation methods was compared by nucleotide composition plots which show the mean base composition for every read cycle of NGS and indicate—at the beginning of the reads—the quality of the random fragmentation (Fig 3A). The difference between the mean base composition for every read cycle and the average base composition in the reads was estimated using the chi-squared test (Fig 3B).

The deviations of the plots from the average base composition in the first three positions of the reads (Fig 3A) and the increased chi-square value at the first positions of the reads (Fig 3B) indicate that the sites of DNA fragmentation for both enzymatic methods are partly associated with DNA sequence contents. This is no surprise because all methods of fragmentation are partly sequence-specific [2, 6, 10, 11]. We expected a lower randomization of FTP DNA digestion in comparison with Fragmentase because DNase I—used in FTP—is a sequence-dependent enzyme [8, 9]. However, the FTP method provided the better randomization of the fragmentation sites than Fragmentase (Fig 3). Perhaps the generation of overlapping sequences at the ends of FTP fragments (Fig 1) counterbalances the sequence-dependent DNA nicking by DNase I.

**Fig 4:**
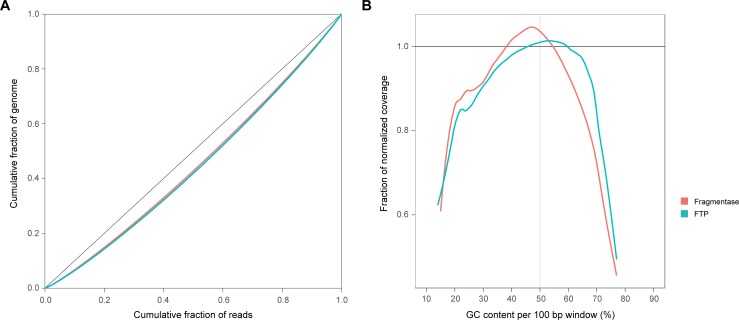
Coverage uniformity evaluation. Cumulative read coverage was visualized as Lorenz curves (A) and GC bias of the coverage was estimated as normalized coverage over GC content for both Fragmentase (red curves) and FTP (blue curves) methods. **(A)** The Lorenz curves show the cumulative fraction of the genome as a function of the cumulative fraction of the reads. Perfectly uniform coverage would result in a diagonal line (black). Fragmentase and FTP methods exhibit the same deviations from the diagonal as a result of biased coverage. **(B)** The GC bias plots show the normalized coverage as a function of GC content. The black horizontal line (normalized coverage = 1) represents an ideally uniform coverage and any divergence from it indicates either oversequencing (normalized coverage > 1) or underrepresentation (normalized coverage < 1) of the sequences of particular GC content. Both methods give similar uniformity, while FTP provides better coverage for GC reach (> 55% GC content) sequences.

For the efficient and complete extraction of information from the NGS assay, the full and uniform representation of the whole genome sequence in the NGS library is essential. Among other factors, this heavily depends on the level of randomization during the fragmentation step of the library preparation. To assess the representation of the sequences in the FTP and Fragmentase libraries, we visualized the read coverage uniformity over the genome (Fig 4A) and GC coverage bias (Fig 4B) for both methods. As the reference sequence, the *E*.*coli* BL21(DE3) genome sequence (NCBI Ref Seq: NC_012971.2) was used.

To evaluate the read coverage uniformity throughout the genome, Lorenz curves were used. A Lorenz curve shows the cumulative fraction of reads as a function of the cumulative fraction of the genome. The plotted curves (Fig 4A) demonstrate that both the Fragmentase and FTP methods exhibit the same uniformity.

GC coverage bias plots allow the evaluation of the read coverage depending on GC content. A normalized (relative) coverage in the plots is a relative measure of sequence coverage by the reads at a particular GC content. The plot visualizes the normalized coverage across the entire GC spectrum by grouping all 100-base sliding windows across the genome by their GC content and reporting the average normalized coverage for each GC content percentage. A normalized coverage of 1 indicates that a particular base is covered at the expected average rate. A relative coverage above 1 indicates higher than expected coverage and below 1 indicates lower than expected coverage. The obtained GC bias plots (Fig 4B) demonstrate similar uniform coverage depending on GC content, while FTP provides better uniform coverage for GC reach sequences.

There are several key characteristics of NGS that depend on the quality of the library: genome coverage, identity with a reference sequence, the rate of errors, and the number of unmappable sequences. These characteristics were estimated for different sequencing depths of the NGS libraries. For the simulation of different depths, random samples of NGS reads were generated. To compare the genome coverage (the total number of aligned bases in the reference divided by the genome size), we used the genome sequence NCBI Ref Seq: NC_012971.2 as the reference with the assumption that this represented 100% coverage. For the computation of genome coverage, a base in the reference genome is counted as aligned if there is at least one contig with at least one alignment to this base. Contigs from repeat regions may map to multiple places and thus may be counted multiple times in this quantity. Unmappable sequences were calculated as a rate of unmappable reads. A large fraction of these reads would reduce the efficiency and the apparent coverage of the genome sequencing. The rate of indels was estimated as the average number of single nucleotide insertions or deletions per 100,000 aligned bases, and the rate of mismatches was estimated as the average number of mismatches per 100,000 aligned bases. The resulting average data from the NGS analyses are shown in Table 1. The statistics for the Fragmentase and FTP NGS libraries were calculated from the data of the four independent libraries for each fragmentation method. The detailed data for each NGS library are shown in the Supporting information (S1 Table). The obtained characteristics are identical or very similar for the assembled sequences from the libraries generated by the different methods (Table 1). The FTP method gives a greater proportion of unmappable reads compared to Fragmentase, but the difference is less than 1% of all reads in the library. It can be explained by the assumption that FTP generates additional non-specific sequences during the polymerization stage of the fragmentation. Potentially, FTP may increase the level of mismatches, because SD polymerase does not have proofreading activity. In practice, we did not see any significant difference between the methods. Proportions of FTP/Fragmentase mismatches are equal 1 for deep sequencing and 1.08 for shallow (3× depth) sequencing.

**Table 1:** Key averaged NGS characteristics of Fragmentase- and FTP- generated libraries.

Sequencing depth (number of reads)	Method of DNA fragmentation	Genome coverage (%)	Ref. Seq. identity (%)	Mismatch errors (per 100 kb)	Indel errors (per 100 kb)	Unmappable reads (%)
32× depth (10×10^5^ reads)	Fragmentase	98.226	99.999	1.01	0.24	3.07
	FTP	98.224	99.999	1.02	0.14	3.91
16× depth (5×10^5^ reads)	Fragmentase	98.193	99.999	1.05	0.13	3.09
	FTP	98.200	99.999	1.17	0.16	3.92
8× depth (2.5×10^5^ reads)	Fragmentase	98.042	99.996	3.70	0.22	3.17
	FTP	98.068	99.996	4.02	0.24	3.90
3× depth (1×10^5^ reads)	Fragmentase	91.100	99.974	25.23	0.70	3.13
	FTP	90.908	99.971	27.70	1.21	3.90

The mean NGS statistics per library were calculated from the data of the four independent libraries for the each method. All metrics were obtained for different depths of *E*. *coli* BL21 genome sequencing. We found no significant differences between Fragmentase- and FTP- generated NGS libraries.

To evaluate the *de novo* genome assembly of the Fragmentase and FTP libraries, we used QUAST software (quality assessment tool for genome assemblies) [15]. We compared the following assembling metrics:

- Number of contigs: the total number of contigs in the assembly.
- Largest contig: the length of the largest contig in the assembly.
- Total length: the total number of bases in the assembly.
- N50 and N75: the contig length such that using equal or longer length contigs produces at least 50% and 75% (respectively) of the bases of the assembly length [15, 17, 18].
- NG50 and NG75: the contig length such that using equal or longer length contigs produces at least 50% and 75% (respectively) of the length of the reference genome, rather than 50% and 75% of the assembly length [15, 17, 18].

The assembly metrics were calculated for different sequencing depths of the libraries obtained with the Fragmentase and FTP methods. The mean statistics calculated from the data of the four independent libraries for each fragmentation method are shown in Table 2. The metrics for each NGS library are shown in the Supporting information (S2 Table). Our results demonstrate that the characteristics of the genome assembly of the libraries obtained by the novel FTP method are similar to those obtained by the Fragmentase method (Table 2). Fragmentase gives slightly better N50 and N75 metrics for 3× and 8× sequencing depths than FTP, but the difference is not significant because proportions of N50, NG50, N75, NG75 at 3× sequencing depth between Fragmentase and FTP are equal to 1.07–1.09 (close to 1). For deep NGS sequencing (16× and 32× depths), FTP gives the same or slightly better N50 and N75 metrics when compared to Fragmentase.

**Table 2:** The averaged assembly metrics of the NGS libraries obtained by Fragmentase and FTP methods.

Sequencing depth (number of reads)	Method of DNA fragmentation	Number of contigs	Largest contig (bp)	Total length (bp)	N50	NG50	N75	NG75
32× depth (10×10^5^ reads)	Fragmentase	182	272230	4485104	81481	80813	41851	40741
	FTP	195	265892	4484951	81981	80479	43990	41542
16× depth (5×10^5^ reads)	Fragmentase	204	217222	4483651	69766	69259	37530	36159
	FTP	196	194626	4484098	70654	69018	39773	39008
8× depth (2.5×10^5^ reads)	Fragmentase	304	134506	4478279	45010	44221	26078	24944
	FTP	274	133551	4479908	41368	40106	21611	19769
3× depth (1×10^5^ reads)	Fragmentase	2414	14250	4178082	2886	2689	1753	1476
	FTP	2500	15256	4178040	2666	2456	1628	1348

The mean assembly statistics were calculated from the data of the four independent libraries for each method and for the different depths of the *E*. *coli* BL21 genome sequencing. No significant differences between Fragmentase- and FTP- generated NGS libraries were found.

In summary, the Fragmentation Through Polymerization method is a novel, robust, and simple method of DNA fragmentation which is suitable for NGS. In comparison with Fragmentase, it provides very similar characteristics for NGS libraries. Potential disadvantages of FTP are associated with biases of the enzymes used in the method, such as non-random DNA fragmentation and mismatch errors. These characteristics of FTP were compared with the Fragmentase method. The experimental data demonstrate that FTP yields higher quality random fragmentations (Fig 3) and better coverage of GC reach contents (Fig 4B) than Fragmentase. Levels of mismatch errors are similar for both methods. FTP generates a greater number of unmappable reads than Fragmentase, but the difference is less than 1% of all reads in the library.

The main advantage of the FTP method lies in the simplification of NGS library preparation by eliminating the DNA end repair and A-tailing stage from the protocol. In the result, the work time of the procedure can be decreased from 180 minutes to 110 minutes (the repair/A-tailing stage takes 70 minutes according to the manual). Additionally, it can reduce the price of the library preparation. For example, the current price of the NEBNext Ultra II DNA Library Prep kit for 24 reactions is 535 Euros; the price of the NEBNext Ultra II End Repair/dA-Tailing Module for 24 reactions is 262 Euros. Thus, the elimination of this module from the kit can decrease the primary cost of NGS library preparation.

Based on our data we hope that the FTP method can become a helpful tool for NGS.

## Supporting information

S1 Table **Key NGS characteristics of individual libraries generated by Fragmentase (A) and FTP (B) methods.** All metrics were obtained for different depths of *E*. *coli* BL21 genome sequencing.
(DOC)

S2 Table The assembly metrics of the individual NGS libraries obtained by Fragmentase and FTP methods. All metrics were obtained for different depths of *E*. *coli* BL21 genome sequencing.
(DOC)

S1 Fig Optimization of FTP conditions to generate DNA fragments with an optimal average size. **(A)** FTP reactions were performed as described in the Materials and Methods with the different concentrations of DNase I in the reaction mixtures. The obtained DNA fragments were analyzed by agarose-gel electrophoresis. The mixtures contained the following concentrations of DNase I: 1 ng/μl (line 1); 1.5 ng/μl (line 2); 1.875 ng/μl (line 3); 2.25 ng/μl (line 4). M: 100 bp DNA Ladder. Concentration 1 ng/μl of DNase I (line 1) provided the targeted average size (400–600 bp) of the fragments.
**(B)** FTP reactions were performed as described in the Materials and Methods with the different times of incubation at 30°C. The following times were used for the incubation: 10 min. (line 1); 20 min. (line 2); 45 min. (line 3). M: 100 bp DNA Ladder. The incubation at 30°C for 20 minutes (line 2) provided the targeted average size of the fragments (400–600 bp).
(TIF)

S2 Fig Comparison of the sequence qualities scores (PHRED) at the 38-ends of the sequences that have been generated from the NGS libraries constructed with the Fragmentase (red) and FTP (blue) methods of DNA fragmentation. No differences were found between the libraries.
(TIF)
