# Supplementary material for: Fragmentation Through Polymerization (FTP): A new method to fragment DNA for next-generation sequencing
Source: PLoS One. 2019 Apr 1;14(4):e0210374. doi: 10.1371/journal.pone.0210374 (PMC6443234; doi:10.1371/journal.pone.0210374)
Supplement: S1 Table — Key NGS characteristics of individual libraries generated by Fragmentase (A) and FTP (B) methods. All metrics were obtained for different depths of E. coli BL21 genome sequencing. (DOC) [file pone.0210374.s001.doc]

**S1 Table. Key NGS characteristics of individual libraries generated by Fragmentase (A) and FTP (B) metods.**

| **(A) Statistics of Fragmentase NGS libraries** | | | | | | |
| --- | --- | --- | --- | --- | --- | --- |
| **Sequencing depth**  **(number of reads)** | **NGS library** | **Genome coverage (%)** | **Ref. Seq. identity (%)** | **Mismatch errors (per 100 kb)** | **Indel errors (per 100 kb)** | **Unmappable reads (%)** |
| 32× depth  (10×10^5^ reads) | Fragmentase 1 | 98.226 | 99.999 | 0.76 | 0.16 | 3.03 |
|  | Fragmentase 2 | 98.243 | 99.999 | 0.85 | 0.25 | 3.00 |
|  | Fragmentase 3 | 98.226 | 99.999 | 0.67 | 0.25 | 3.58 |
|  | Fragmentase 4 | 98.207 | 99.998 | 1.76 | 0.29 | 2.68 |
|  | Mean per library | **98.226** | **99.999** | **1.01** | **0.24** | **3.07** |
| 16× depth  (5×10^5^ reads) | Fragmentase 1 | 98.184 | 99.999 | 0.89 | 0.11 | 3.03 |
|  | Fragmentase 2 | 98.202 | 99.999 | 0.78 | 0.13 | 2.98 |
|  | Fragmentase 3 | 98.240 | 99.999 | 1.30 | 0.13 | 3.59 |
|  | Fragmentase 4 | 98.146 | 99.999 | 1.25 | 0.16 | 2.71 |
|  | Mean per library | **98.193** | **99.999** | **1.05** | **0.13** | **3.09** |
| 8× depth  (2.5×10^5^ reads) | Fragmentase 1 | 98.155 | 99.996 | 3.60 | 0.29 | 3.03 |
|  | Fragmentase 2 | 98.146 | 99.996 | 3.58 | 0.27 | 3.01 |
|  | Fragmentase 3 | 98.179 | 99.997 | 2.95 | 0.16 | 3.96 |
|  | Fragmentase 4 | 97.690 | 99.995 | 4.67 | 0.16 | 2.69 |
|  | Mean per library | **98.042** | **99.996** | **3.70** | **0.22** | **3.17** |
| 3× depth  (1×10^5^ reads) | Fragmentase 1 | 93.745 | 99.976 | 23.14 | 0.54 | 3.13 |
|  | Fragmentase 2 | 91.644 | 99.971 | 28.03 | 0.55 | 3.05 |
|  | Fragmentase 3 | 93.601 | 99.974 | 24.61 | 1.05 | 3.61 |
|  | Fragmentase 4 | 85.409 | 99.974 | 25.12 | 0.64 | 2.73 |
|  | Mean per library | **91.100** | **99.974** | **25.23** | **0.70** | **3.13** |

| **(B) Statistics of FTP NGS libraries** | | | | | | |
| --- | --- | --- | --- | --- | --- | --- |
| **Sequencing depth**  **(number of reads)** | **NGS library** | **Genome coverage (%)** | **Ref. Seq. identity (%)** | **Mismatch errors (per 100 kb)** | **Indel errors (per 100 kb)** | **Unmappable reads (%)** |
| 32× depth  (10×10^5^ reads) | FTP 1 | 98.227 | 99.999 | 0.67 | 0.18 | 3.97 |
|  | FTP 2 | 98.235 | 99.999 | 1.36 | 0.13 | 3.71 |
|  | FTP 3 | 98.213 | 99.999 | 1.23 | 0.16 | 3.97 |
|  | FTP 4 | 98.219 | 99.999 | 0.83 | 0.07 | 3.98 |
|  | Mean per library | **98.224** | **99.999** | **1.02** | **0.14** | **3.91** |
| 16× depth  (5×10^5^ reads) | FTP 1 | 98.220 | 99.999 | 1.00 | 0.18 | 3.96 |
|  | FTP 2 | 98.195 | 99.998 | 1.76 | 0.22 | 3.72 |
|  | FTP 3 | 98.190 | 99.999 | 0.67 | 0.11 | 3.96 |
|  | FTP 4 | 98.194 | 99.999 | 1.25 | 0.11 | 4.02 |
|  | Mean per library | **98.200** | **99.999** | **1.17** | **0.16** | **3.92** |
| 8× depth  (2.5×10^5^ reads) | FTP 1 | 98.159 | 99.996 | 3.26 | 0.09 | 3.93 |
|  | FTP 2 | 98.012 | 99.995 | 4.54 | 0.34 | 3.73 |
|  | FTP 3 | 98.024 | 99.996 | 4.21 | 0.13 | 3.97 |
|  | FTP 4 | 98.075 | 99.996 | 4.05 | 0.40 | 3.99 |
|  | Mean per library | **98.068** | **99.996** | **4.02** | **0.24** | **3.90** |
| 3× depth  (1×10^5^ reads) | FTP 1 | 91.257 | 99.971 | 27.57 | 0.94 | 3.94 |
|  | FTP 2 | 90.615 | 99.971 | 27.72 | 1.57 | 3.71 |
|  | FTP 3 | 91.340 | 99.971 | 27.78 | 1.15 | 3.97 |
|  | FTP 4 | 90.418 | 99.971 | 27.73 | 1.19 | 3.96 |
|  | Mean per library | **90.908** | **99.971** | **27.70** | **1.21** | **3.90** |
