# Supplementary material for: Fragmentation Through Polymerization (FTP): A new method to fragment DNA for next-generation sequencing
Source: PLoS One. 2019 Apr 1;14(4):e0210374. doi: 10.1371/journal.pone.0210374 (PMC6443234; doi:10.1371/journal.pone.0210374)
Supplement: S2 Table — All metrics were obtained for different depths of E. coli BL21 genome sequencing. (DOC) [file pone.0210374.s002.doc]

**S2 Table. The assembly metrics of the individual NGS libraries obtained by Fragmentase and FTP methods.**

| **(A) Assembly metrics of Fragmentase NGS libraries** | | | | | | | | |
| --- | --- | --- | --- | --- | --- | --- | --- | --- |
| **Sequencing depth**  **(number of reads)** | **NGS library** | **Number of contigs** | **Largest contig (bp)** | **Total length (bp)** | **N50** | **NG50** | **N75** | **NG75** |
| 32× depth  (10×10^5^ reads) | Fragmentase 1 | 176 | 295511 | 4485053 | 75532 | 75532 | 40065 | 39754 |
|  | Fragmentase 2 | 180 | 295511 | 4486136 | 77126 | 75532 | 44129 | 42969 |
|  | Fragmentase 3 | 186 | 295509 | 4485494 | 86633 | 86633 | 42969 | 40121 |
|  | Fragmentase 4 | 184 | 202390 | 4483732 | 86633 | 85554 | 40240 | 40121 |
|  | Mean per library | **182** | **272230** | **4485104** | **81481** | **80813** | **41851** | **40741** |
| 16× depth  (5×10^5^ reads) | Fragmentase 1 | 203 | 236222 | 4483526 | 75532 | 74058 | 40065 | 39754 |
|  | Fragmentase 2 | 199 | 295509 | 4484059 | 68738 | 68738 | 39231 | 37299 |
|  | Fragmentase 3 | 175 | 202476 | 4484984 | 85554 | 85554 | 46104 | 43904 |
|  | Fragmentase 4 | 238 | 134680 | 4482033 | 49239 | 48686 | 24719 | 23677 |
|  | Mean per library | **204** | **217222** | **4483651** | **69766** | **69259** | **37530** | **36159** |
| 8× depth  (2.5×10^5^ reads) | Fragmentase 1 | 211 | 169496 | 4482573 | 56777 | 55131 | 33270 | 32036 |
|  | Fragmentase 2 | 227 | 150822 | 4482514 | 48318 | 47132 | 27213 | 24741 |
|  | Fragmentase 3 | 214 | 167691 | 4482967 | 59598 | 59411 | 35163 | 34861 |
|  | Fragmentase 4 | 564 | 50015 | 4465061 | 15345 | 15211 | 8666 | 8137 |
|  | Mean per library | **304** | **134506** | **4478279** | **45010** | **44221** | **26078** | **24944** |
| 3× depth  (1×10^5^ reads) | Fragmentase 1 | 1943 | 18622 | 4300533 | 3460 | 3283 | 2034 | 1811 |
|  | Fragmentase 2 | 2413 | 15114 | 4202007 | 2813 | 2640 | 1713 | 1394 |
|  | Fragmentase 3 | 1882 | 14113 | 4292083 | 3450 | 3256 | 2094 | 1854 |
|  | Fragmentase 4 | 3420 | 9149 | 3917707 | 1819 | 1578 | 1170 | 844 |
|  | Mean per library | **2414** | **14250** | **4178082** | **2886** | **2689** | **1753** | **1476** |

| **(B) Assembly metrics of FTP NGS libraries** | | | | | | | | |
| --- | --- | --- | --- | --- | --- | --- | --- | --- |
| **Sequencing depth**  **(number of reads)** | **NGS library** | **Number of contigs** | **Largest contig (bp)** | **Total length (bp)** | **N50** | **NG50** | **N75** | **NG75** |
| 32× depth  (10×10^5^ reads) | FTP 1 | 204 | 295511 | 4484886 | 85298 | 85298 | 45506 | 43998 |
|  | FTP 2 | 182 | 295509 | 4485396 | 85554 | 85554 | 42854 | 40121 |
|  | FTP 3 | 183 | 170365 | 4484942 | 75532 | 75532 | 44159 | 41810 |
|  | FTP 4 | 210 | 302183 | 4484579 | 81541 | 75532 | 43441 | 40240 |
|  | Mean per library | **195** | **265892** | **4484951** | **81981** | **80479** | **43990** | **41542** |
| 16× depth  (5×10^5^ reads) | FTP 1 | 193 | 295511 | 4485056 | 75532 | 75532 | 43995 | 42969 |
|  | FTP 2 | 200 | 164284 | 4483586 | 72482 | 71899 | 39231 | 39189 |
|  | FTP 3 | 193 | 161580 | 4484052 | 65727 | 59904 | 36687 | 35163 |
|  | FTP 4 | 197 | 157129 | 4483699 | 68875 | 68738 | 39177 | 38710 |
|  | Mean per library | **196** | **194626** | **4484098** | **70654** | **69018** | **39773** | **39008** |
| 8× depth  (2.5×10^5^ reads) | FTP 1 | 243 | 156884 | 4483588 | 45082 | 42659 | 25814 | 20336 |
|  | FTP 2 | 298 | 128426 | 4477069 | 40206 | 39947 | 20336 | 19478 |
|  | FTP 3 | 270 | 115888 | 4478286 | 41781 | 39650 | 20901 | 20442 |
|  | FTP 4 | 286 | 133007 | 4480688 | 38404 | 38167 | 19394 | 18819 |
|  | Mean per library | **274** | **133551** | **4479908** | **41368** | **40106** | **21611** | **19769** |
| 3× depth  (1×10^5^ reads) | FTP 1 | 2507 | 24625 | 4193948 | 2657 | 2468 | 1610 | 1364 |
|  | FTP 2 | 2511 | 11780 | 4163190 | 2630 | 2417 | 1626 | 1330 |
|  | FTP 3 | 2329 | 11990 | 4200510 | 2898 | 2651 | 1755 | 1452 |
|  | FTP 4 | 2654 | 12628 | 4154512 | 2480 | 2282 | 1520 | 1247 |
|  | Mean per library | **2500** | **15256** | **4178040** | **2666** | **2456** | **1628** | **1348** |
