# Supplementary figures and images for: Fragmentation Through Polymerization (FTP): A new method to fragment DNA for next-generation sequencing
Source: PLoS One. 2019 Apr 1;14(4):e0210374. doi: 10.1371/journal.pone.0210374 (PMC6443234; doi:10.1371/journal.pone.0210374)

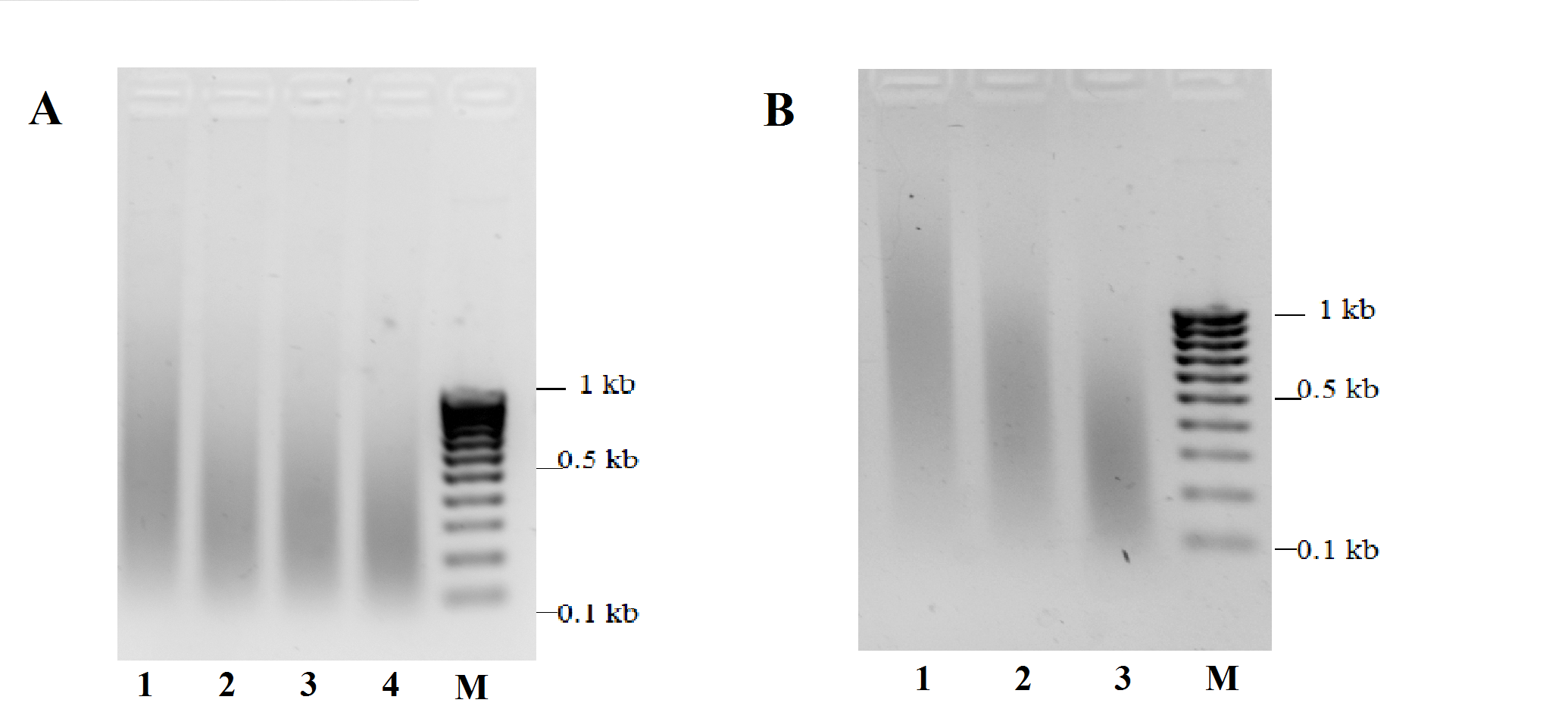

Supplement: S1 Fig — (A) FTP reactions were performed as described in the Materials and Methods with the different concentrations of DNase I in the reaction mixtures. The obtained DNA fragments were analyzed by agarose-gel electrophoresis. The mixtures contained the following concentrations of DNase I: 1 ng/μl (line 1); 1.5 ng/μl (line 2); 1.875 ng/μl (line 3); 2.25 ng/μl (line 4). M: 100 bp DNA Ladder. Concentration 1 ng/μl of DNase I (line 1) provided the targeted average size (400–600 bp) of the fragments. (B) FTP reactions were performed as described in the Materials and Methods with the different times of incubation at 30°C. The following times were used for the incubation: 10 min. (line 1); 20 min. (line 2); 45 min. (line 3). M: 100 bp DNA Ladder. The incubation at 30°C for 20 minutes (line 2) provided the targeted average size of the fragments (400–600 bp). (TIF) [file pone.0210374.s003.tif]

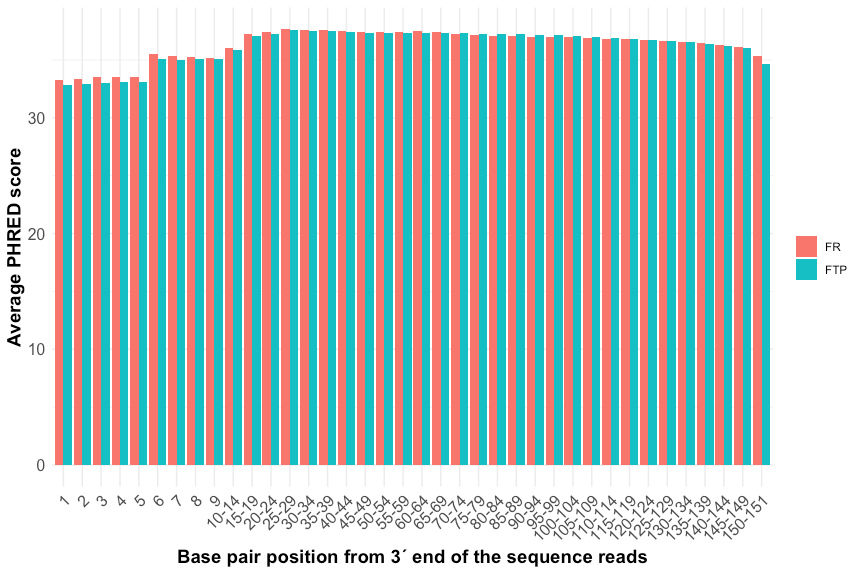

Supplement: S2 Fig — No differences were found between the libraries. (TIF) [file pone.0210374.s004.tif]
